# Synchronous childhood T-lymphoblastic lymphoma and B-cell precursor acute lymphoblastic leukemia associated with constitutional MMR deficiency syndrome –a case report

**DOI:** 10.3389/fonc.2025.1580494

**Published:** 2025-05-16

**Authors:** Joanna Bulsa, Agata Karolina Pastorczak, Joanna Taha, Wojciech Mlynarski, Łukasz Sędek, Tomasz Szczepanski

**Affiliations:** ^1^ Medical University of Silesia, Katowice, Poland; ^2^ Medical University of Lodz, Łódź, Poland

**Keywords:** CMMRD, T-LBL, BCP-ALL, genetic cancer predisposition, synchronous cancerogenesis

## Abstract

We present a four-year-old girl with synchronous T-cell lymphoblastic lymphoma (T-LBL) and B-cell precursor acute lymphoblastic leukemia (BCP-ALL).Development of two lymphoid malignancies arising from completely different cell lineages was confirmed by molecular tests. The cause of cancerogenesis in this patient was a genetic predisposition resulting from mismatch repair deficiency syndrome (CMMRD). Herein, we present the fatal course of the disorder, challenging treatment decisions and need for special approach to the patients with CMMRD-associated malignant proliferation.

## Introduction

Lymphoid malignancies are the most common cancers in children and adolescents, with an incidence rate of nearly 40% in this population. Both acute lymphoblastic leukemia (ALL) and T-lymphoblastic lymphoma (T-LBL) represent proliferative disorders arising from aberrant, immature lymphoid precursors. The etiology of childhood neoplasms remains largely unknown. However, several risk factors have been identified, including ionizing radiation, chemicals (such as pesticides, hydrocarbons, and certain therapeutic agents), immunodeficiency disorders, and specific genetic defects (e.g., Down syndrome, Fanconi anemia, Bloom syndrome, Nijmegen syndrome, and ataxia-telangiectasia) (1).

Genetic disorders are often characterized by structural chromosomal abnormalities or faulty DNA repair mechanisms, which may increase the risk of childhood hematological malignancies (1). Moreover, some inherited mutations play a key role in cancer predisposition at a young age (2). Cancer predisposition genes are involved in the dysregulation of DNA damage repair, mitotic cell division, and developmental gene expression (2).

Mutations in genes associated with DNA repair mechanisms can lead to the accumulation of pathological alterations in tumor suppressor genes and oncogenes, which may contribute to early-onset cancer development (2). The mismatch repair (MMR) pathway is responsible for correcting DNA replication errors—such as base pair mismatches and insertion/deletion loops—following DNA synthesis (3–5).

Germline mutations in *MMR* genes, including *MLH1, MSH2, MSH6*, and *PMS2*, are associated with highly microsatellite-unstable tumors (3). Heterozygous germline mutations in MMR genes cause Lynch syndrome, which is associated with hereditary nonpolyposis colorectal cancer (HNPCC), as well as breast and ovarian cancers. It is the most common hereditary cancer predisposition syndrome, inherited in an autosomal dominant manner (3, 4).

In contrast to this well-characterized syndrome, relatively little is known about biallelic, recessively inherited mutations in *MMR* genes that result in the recently identified “mismatch syndrome,” or constitutional mismatch repair deficiency syndrome (CMMRD) (3). This rare disorder is characterized by a high predisposition to hematological malignancies, brain tumors, and bowel tumors at an early age. It is frequently associated with café-au-lait spots and various forms of immunodeficiency (3, 6).

The first cases of this disorder were described in 1959 by Jacques Turcot, involving two siblings with colorectal polyps and carcinomas associated with brain tumors—later known as Turcot syndrome (7). Retrospective molecular analyses have shown that many of the gene alterations in Turcot syndrome overlap with those observed in CMMRD (7).

The simultaneous development of two distinct types of malignancies is extremely rare and may suggest an underlying genetic predisposition, including CMMRD. Herein, we present the case of a patient with mediastinal T-lymphoblastic lymphoma and bone marrow infiltration by B-precursor acute lymphoblastic leukemia (BCP-ALL), in the context of mismatch repair deficiency syndrome.

## Methods

### General diagnostic assay

Multicolor flow cytometry, conventional cytogenetics, fluorescence *in situ* hybridization (FISH), and histopathological evaluation were performed in certified clinical laboratories according to standard protocols.

### Panel-based RNA sequencing

RNA sequencing was performed using the TruSight RNA Pan-Cancer Panel (Illumina, San Diego, CA, USA), which contains 1,385 cancer-related genes and enables fusion calling and variant detection within the panel. A total of 20 ng of RNA was processed according to the manufacturer’s protocol. A pooled cDNA library of the patient sample together with twenty-three other samples was prepared and sequenced on a NextSeq 550 system (Illumina, USA) using the NextSeq^®^ Reagent Kit v3 (150 cycles) with a paired-end NextSeq^®^ Flow Cell.

Data analysis was performed using Illumina BaseSpace applications: TopHat Alignment (version 1.0.0, read mapping on the hg19 reference genome by TopHat2, fusion calling by TopHat-Fusion) and RNA-seq Alignment (version 1.1.0, read mapping on hg19 by STAR, fusion calling by Manta), using default settings (https://basespace.illumina.com/apps).

### Direct sequencing

The presence of a nonsense variant in exon 11 of the PMS2 gene (NM_000535.7: c.1939A>T, p.Lys647Ter) and a stop-loss variant in exon 10 of the PAX5 gene (NM_016734.3: c.1174T>C, p.Ter392Argext*111) was confirmed by direct sequencing. Standard PCR conditions were applied using primers specifically designed to cover exon 11 of PMS2 and exon 10 of PAX5, designed with NetPrimer software. The products were sequenced on an ABI 3130 4-capillary sequencer (Thermo Fisher Scientific, Waltham, USA), and the results were analyzed using Sequencher v5.0 (Gene Codes, Ann Arbor, USA).

## Case presentation

A 4-year-old Polish girl, with a history of refractory middle ear otitis and episodic bronchospasm, was admitted to the Pediatric Hematology and Oncology Department due to a large mediastinal mass. Eight months prior to diagnosis, she had been evaluated for suspected primary immunodeficiency due to recurrent infections and hypogammaglobulinemia, although no definitive diagnosis was established. Five days before admission, she developed symptoms of acute respiratory infection. A chest X-ray revealed a suspicious mediastinal shadow.

On admission, the patient presented with non-productive cough, dyspnea, wheezing, and orthopnea.

Physical examination revealed cyanosis, moderately enlarged cervical and submandibular lymph nodes, decreased vesicular breath sounds over the left lung field, and two café-au-lait (CAL) lesions on the left forearm and anterior abdominal wall. There was no hepatosplenomegaly or other remarkable findings. Her family history was notable for one case of malignant lymphoma and one case of breast cancer.

Blood morphology and peripheral smear were within normal limits. Serum lactate dehydrogenase (LDH) was elevated at 738 U/L. Other biochemical parameters were unremarkable. Contrast-enhanced chest CT revealed a large mediastinal and left thoracic mass displacing mediastinal structures, narrowing the airways, and infiltrating the left pectoralis muscle.

Due to life-threatening symptoms caused by a mediastinal mass of unknown origin, the patient underwent a left posterolateral thoracotomy with excision of approximately 80% of the tumor. The procedure was performed both to obtain tissue for histopathological diagnosis and as a life-saving intervention aimed at relieving severe mediastinal compression.

**Figure 1:**
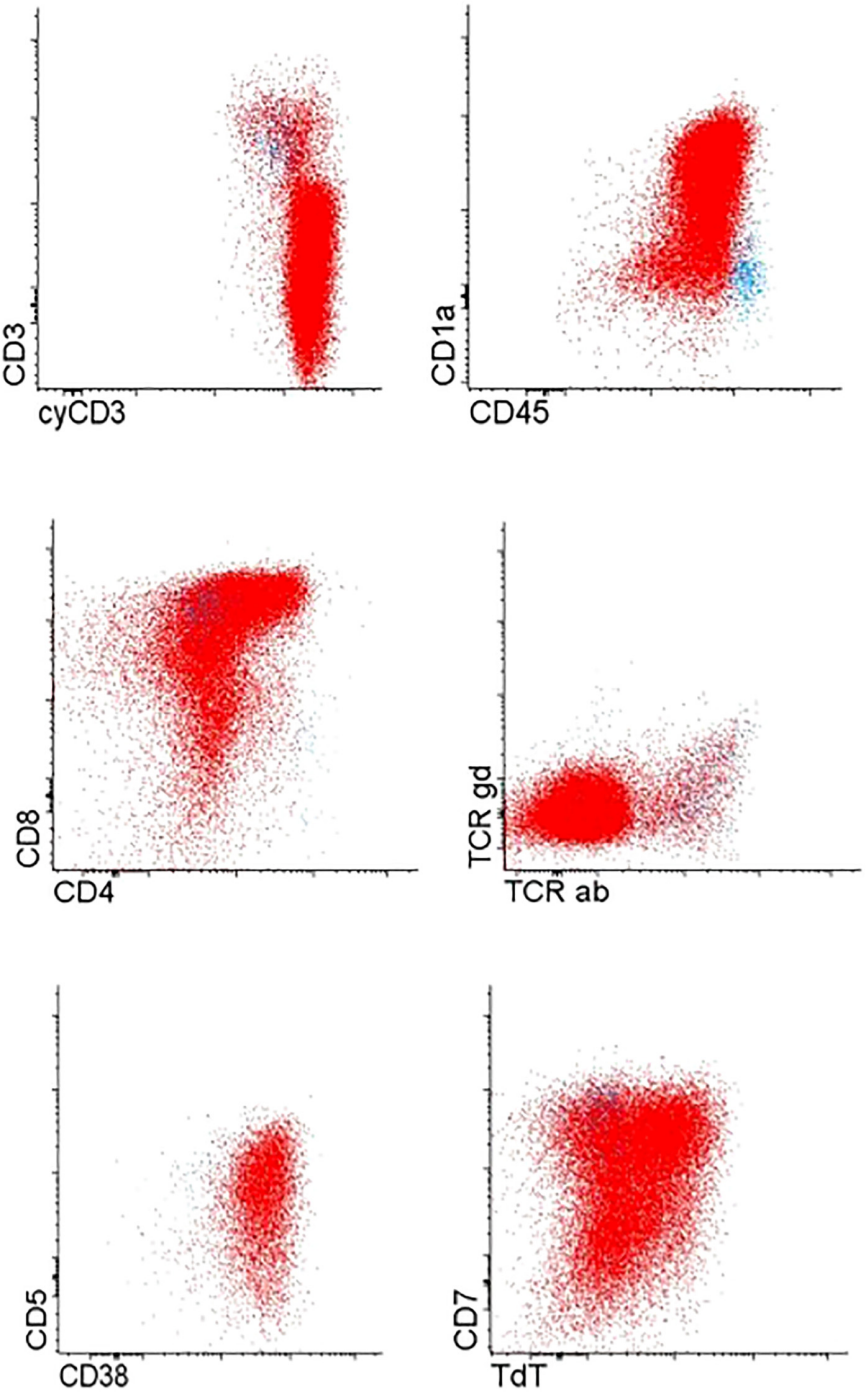
T-cell precursor cells of mediastinal tumor.

**Figure 2:**
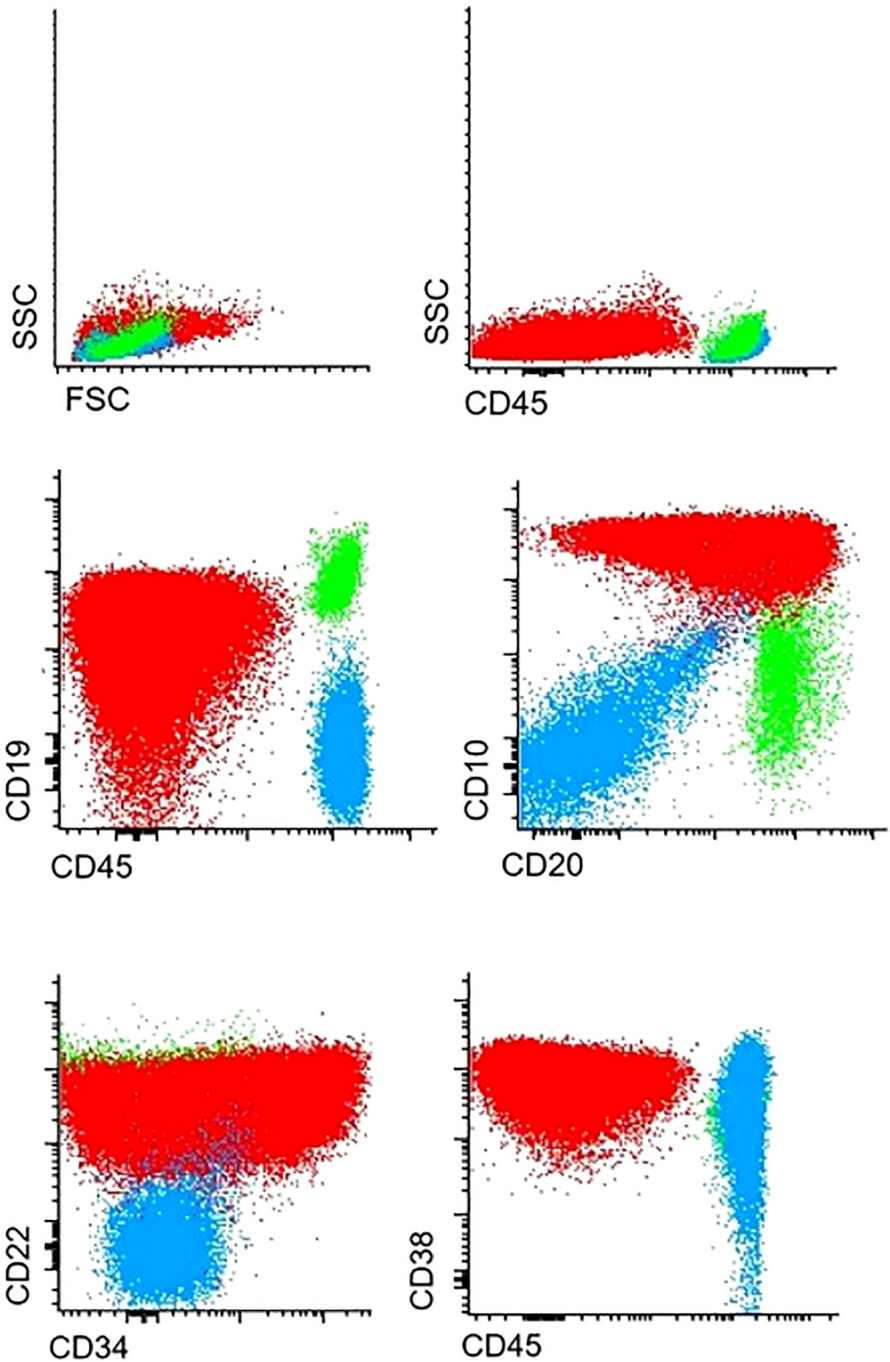
Infiltration of B-cell precursor cells in bone marrow.

Immunophenotyping revealed T cell lymphoblastic lymphoma (T-LBL), with high expression of typical T-markers, such as CD3 and CD7 (**Figure 1**). Histopathological findings confirmed the initial diagnosis of T-LBL. During the routine staging investigation, bone marrow aspiration identified infiltration of 56.8% of B-cell precursors with positive expression of pan B-cell markers such as CD19, CD10, CD79a, and CD20 and negative expression of T-cell markers (**Figure 2**). These findings led to a final diagnosis of mediastinal T-LBL associated with BCP-ALL in the bone marrow.

Taking into account the lymphoid origin of both malignancies, the treatment strategy scheduled chemotherapy according to the current AIEOP-BFM-based treatment protocol. A 33-day follow-up CT of the chest showed almost complete regression in tumor size, and bone marrow evaluation revealed a negative score for molecular minimal residual disease (MRD-PCR). Genetic tests (karyotype, FISH) did not show any recurrent abnormalities including BCR::ABL1, KMT2A, ETV6::RUNX1, or TCF3::PBX1. Thus, a patient was classified as medium risk, the treatment plan assumed further chemotherapy according to AIEOP-BFM backbone (Protocol IB, Protocol M, Protocol II, and maintenance treatment). Chemotherapy was complicated by myelotoxicities and severe infections, including episodes of pneumonia and invasive fungal disease with abscesses in the central nervous system. For this reason, immunoglobulin replacement in combination with antibiotic prophylaxis was applied throughout the treatment period. Moreover, when the patient started maintenance treatment with 6-Mercaptopurine and Methotrexate, facial hyperpigmentation occurred.

One month before the planned end of maintenance therapy, the patient again presented with pneumonia. A chest CT revealed significant tumor regrowth. Open biopsy confirmed isolated relapse of mediastinal T-LBL, while bone marrow remained negative for malignant infiltration. The patient was qualified for the second-line chemotherapy, followed by allogeneic stem cell transplantation (allo HSCT). Initial salvage treatment included cycles of HIA (high-dose cytarabine and idarubicin) and HC1 (high-dose cyclophosphamide-based regimen 1), which provided temporary stabilization. However, tumor progression resumed shortly thereafter.

In the light of poor response to previous therapies and the severity of symptoms (rapid tumor growth, superior vena cava syndrome, dyspnea, wheezing), the treatment strategy was highly controversial. Following multidisciplinary consultation, therapy with nelarabine in combination with cyclophosphamide and etoposide (VP/CPM) was initiated. After two cycles, disease progression continued. Subsequently, high-dose cyclophosphamide was administered without clinical improvement. Due to life-threatening symptoms, the patient received emergency radiotherapy targeting the mediastinal mass.

Over the course of treatment, the patient’s clinical condition continued to worsen: the patient exhibited signs of superior vena cava syndrome (facial, neck, and upper limb swelling) and oxygen-dependent dyspnea. Due to the rapid progression of a highly resistant tumor and the ineffectiveness of treatment, the patient died of respiratory failure seven months after relapse diagnosis.

Further molecular analysis based on genomic DNA sequencing revealed a biallelic nonsense mutation in exon 11 of the *PMS2* gene (NM_000535.7: c.1939A>T, p.Lys647Ter), present in both tumor types and in germline cells. In addition, a stop-loss variant in exon 10 of the *PAX5* gene (NM_016734.3: c.1174T>C, p.Ter392Argext*111) was detected exclusively in leukemic blasts but not in T-LBL or germline cells. Genetic testing confirmed that the patient’s mother was a heterozygous asymptomatic carrier of the *PMS2* mutation (**Table 1**).

**Table 1:** DNA sequencing of the patient and her family.

Patient	Genotype	Variant characteristics	Phenotype
Patient	*PMS2* NM_000535.7:c.1939A>T NP_000526.2:p.Lys647Ter	Homozygous, pathogenic	Symptomatic
Mother	*PMS2* NM_000535.7:c.[1939A>T];[1939=] NP_000526.2:p..[(Lys647Ter)];[(=)]	Heterozygous, potentially pathogenic	Asymptomatic carrier
Father	*PMS2* NM_032638.5:c.[1939A=] NP_000526.2:p.[(Lys647=)]	Normal	Healthy

**Figure 3:**
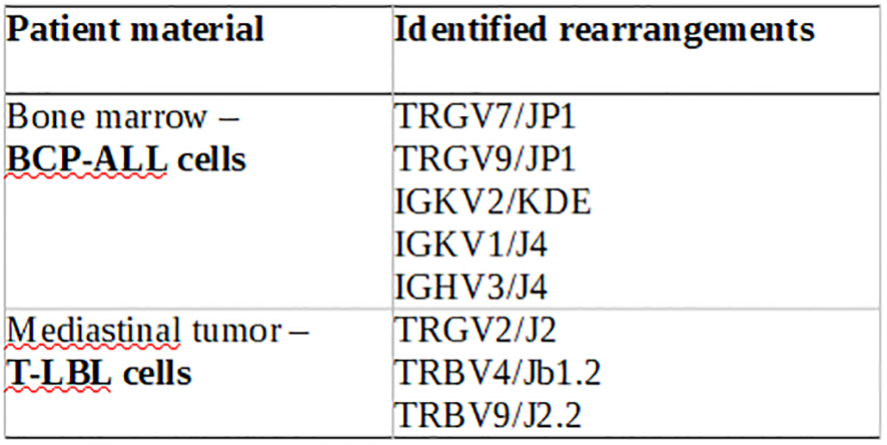
IG/TCR rearragements of the patient material – clonal gene rearrengemnts are completely different between BCP-ALL and T-LBL.

Molecular clonality analysis of IG/TCR gene rearrangements confirmed the independent origins of the two malignancies. In leukemic cells (BCP-ALL), rearrangements included IGKV2/KDE, IGKV1/J4, and IGHV3/J4; in lymphoma cells (T-LBL), the following were observed: TRGV2/J2, TRBV4/JB1.2, and TRBV9/J2.2 (**Figure 3**). This strongly supports the hypothesis of two clonally unrelated neoplastic processes, a highly unusual but clinically important finding in pediatric hematologic malignancies.

## Discussion

The simultaneous presentation of two cancers of distinct origins is extremely rare. To our knowledge, this is the first reported case of synchronous B-cell precursor acute lymphoblastic leukemia (BCP-ALL) and T-lymphoblastic lymphoma (T-LBL). Based on previous studies, the coexistence of biologically unrelated malignancies may be the result of underlying conditions, including primary immunodeficiencies or specific genetic alterations (8, 9). Existing literature has documented the co-occurrence of colorectal adenocarcinomas with other epithelial tumors in Lynch syndrome. However, to date, no reports have described the presence of two entirely distinct lymphoproliferative neoplasms, particularly in a pediatric patient. In the present case, the diagnosis of two independent malignancies was confirmed by both immunophenotyping and molecular testing. Clonality analysis of IG/TCR gene rearrangements revealed two completely distinct neoplastic clones.

In our case, constitutional mismatch repair deficiency syndrome (CMMRD) was genetically confirmed. A mutation in the mismatch repair (MMR) gene family was detected in both malignancies (T-LBL and BCP-ALL) as well as in germline cells. Notably, *PAX5* has been identified as a downstream target in *MMR*-deficient ALL and plays a key role in leukemogenesis by disrupting the regulation of lymphoid transcription factors (5). In a large cohort of relapsed pediatric ALL, Best et al. showed that *MMR*-deficient cells can become immortalized, acquiring a high potential for secondary mutations due to a loss of senescence and unrestricted self-renewal (5). The presence of a *PAX5* mutation in this case may have played a critical role in the leukemic transformation of *MMR*-deficient cells.

It is well established that patients with CMMRD are at increased risk of developing malignant lymphoid proliferations (9, 10). Approximately one-third of patients with CMMRD develop leukemia or lymphoma, with a median age of onset of six years (10). The most commonly reported malignancies include T-cell lymphoblastic lymphoma, Burkitt lymphoma, diffuse large B-cell lymphoma (DLBCL), and acute lymphoblastic leukemia (10). Among these, T-cell precursor malignancies, particularly T-LBL, are most frequent (9, 10).

Despite the growing recognition of CMMRD-related malignancies, the clinical behavior of these cancers remains poorly understood. Several case reports have highlighted an aggressive disease course, frequent relapses, increased toxicity related to chemotherapy, and the development of secondary malignancies (5, 10). Ripperger et al. reported a high relapse rate in MMR-deficient ALL (10), while similar outcomes have been described in T-LBL associated with CMMRD. In our case, the disease course was aggressive, evidenced by the simultaneous onset of two distinct lymphoid malignancies and early relapse of T-LBL, occurring just 23 months after the initial diagnosis. The relapsed T-LBL was highly progressive and resistant to both chemotherapy and radiotherapy.

*MMR-*deficiency is one potential mechanism underlying multidrug resistance (11). The *MMR* pathway plays a central role in recognizing and correcting DNA polymerase errors—such as base substitutions, deletions, and insertions—and initiating subsequent DNA repair or apoptosis (11). Structural DNA alterations may also result from the action of certain chemotherapeutic agents. DNA adducts formed by methylating or alkylating agents are normally detected by the *MMR* complex, which then activates apoptotic pathways. However, in *MMR*-deficient cells, this recognition and repair process is impaired, allowing continued proliferation with an accumulation of mutagenic lesions and a higher mutation rate (11, 12).

Studies have shown that CMMRD is associated with resistance to numerous cytotoxic drugs, including methylating agents, thiopurines, platinum compounds, and topoisomerase II inhibitors (11). Exposure of *MMR*-deficient cells to antimetabolites may further increase mutagenesis, thereby contributing to relapse or rapid tumor progression (12). In our case, *MMR-*deficiency likely influenced both disease aggressiveness and treatment response. Therefore, the presence of *MMR* gene mutations should be regarded as an unfavorable prognostic factor due to their association with drug resistance and high mutational burden.

Timely detection of CMMRD mutations in patients with lymphoproliferative disorders is essential and has significant clinical implications, including considerations for chemotherapy dose adjustments, extended recovery periods, prophylactic strategies for managing immunosuppression, and close monitoring of organ function. Unfortunately, due to nonspecific clinical features, diagnosis is often delayed. To improve early detection, the European consortium ‘Care for CMMRD’ proposed a scoring system to identify candidates for molecular testing (**Table 2**) (7). This includes a broad spectrum of malignancies, phenotypic features (e.g., NF1-like manifestations or pigmentation abnormalities), and serum immunoglobulin levels (e.g., IgG2/4 or IgA deficiency) (7, 13). Developing diagnostic algorithms based on phenotypic and oncologic criteria may facilitate early identification of patients at risk for CMMRD and support the design of individualized surveillance plans (13).

**Table 2:** C4CMMRD scoring system for a clinical suspicion of CMMRD in cancer patients (13).

Indication for CMMRD testing in cancer patients	>3 points
Malignancies/premalignancies: one is mandatory; if more than one is present in the patient, add the points:	
Carcinoma from the LS spectrum at age <25 years	3 points
Multiple bowel adenomas at age <25 years and absence of *APC/MUTYH* mutation(s) or a single high-grade dysplasia adenoma at age <25 years	3 points
WHO grade III or IV glioma at age <25 years	2 points
NHL of T-cell lineage or sPNET at age <18 years	2 points
Any malignancy at age <18 years	1 point
Additional features: optional; if more than one of the following is present, add the points	
Clinical sign of NF1 and/or 2 hyperpigmented and/or hypopigmented skin alterations >1 cm in the patient	2 points
Diagnosis of LS in a first-degree or second-degree relative	2 points
Carcinoma from LS spectrum a before the age of 60 in first-degree, second-degree, or third-degree relative	1 point
A sibling with carcinoma from the LS spectrum, high-grade glioma, sPNET, or NHL	2 points
A sibling with any type of childhood malignancy	1 point
Multiple pilomatricomas in the patient	2 points
One pilomatricoma in the patient	1 point
Agenesis of the corpus callosum or non-therapy–induced cavernoma in the patient	1 point
Consanguineous parents	1 point
Deficiency/reduced levels of IgG2/4 and/or IgA	1 point

Identifying CMMRD is also critical for family screening and risk assessment (14). The syndrome, resulting from biallelic mutations in MMR genes, is inherited in an autosomal recessive pattern (4). The diagnosis should prompt genetic testing of family members. In the present case, a potentially pathogenic heterozygous *PMS2* variant was identified in the patient’s mother, who remained asymptomatic at age 45. Testing of family members is essential to inform counseling, surveillance, and preventive strategies.

Beyond the diagnosis, selecting the optimal treatment for *MMR-*deficient patients remains a major challenge. Therapeutic data are still limited. Available reports suggest frontline regimens with reduced toxicity, avoiding methylating agents, thiopurines, and topoisomerase II inhibitors (11, 12). Moreover, allogeneic stem cell transplantation at first remission is a promising option for relapse prevention, which is usually associated with an adverse prognosis in CMMRD (10, 15).

Emerging data on immunotherapy are encouraging. Studies in Lynch syndrome patients have demonstrated favorable outcomes with immune checkpoint inhibitors (16). *MMR*-deficient colorectal cancers often overexpress immunomodulatory proteins and exhibit high sensitivity to PD-1 blockade (17). Several case reports have documented durable responses to nivolumab in patients with biallelic *MMR*-deficient glioblastoma (17, 18). Whether patients with other *MMR*-deficient malignancies will benefit similarly remains under investigation.

Another potential strategy for cancer prevention in this population is low-dose acetylsalicylic acid (ASA). Leenders et al. reviewed the role of ASA in reducing colorectal cancer risk in Lynch syndrome, likely via COX-1–mediated antiplatelet activity (19). While promising, the impact of ASA on cancer mortality remains controversial, and further studies are needed to evaluate its short- and long-term efficacy and safety in preventing relapse of CMMRD-related T-LBL (19).

In the presented patient, the selection of the first-line therapy was based on presence of leukemic infiltration in the bone marrow. The presence of more than 25% blasts in the bone marrow qualifies the patient for treatment according to the AIEOP BFM protocol. Given the early remission and molecular MRD negativity, the patient was stratified into the intermediate-risk group, in accordance with protocol criteria. Due to the favorable molecular response to initial treatment, the AIEOP BFM protocol does not include allogeneic hematopoietic stem cell transplantation, even if the *MMR* mutation status had been known at that time.

During treatment, adverse effects such as myelotoxicity were observed, although these did not exceed the expected range of hematologic complications associated with intensive chemotherapy. Recurrent infections were likely related to an underlying constitutional immunodeficiency, previously suspected prior to diagnosis. However, none of the infectious episodes met the criteria for severe or life-threatening complications, and therefore no dose reductions were required. As part of supportive care, the patient received immunoglobulin supplementation and prophylactic antibiotic therapy, which enabled uninterrupted continuation of treatment without the need for protocol modification.

## Conclusions

The coexistence of two biologically distinct malignancies is extremely rare. Any atypical tumor combination, early-onset malignancy, or development of multiple neoplasms should raise suspicion for an underlying hereditary cancer predisposition syndrome. Although rare, CMMRD is a high-risk condition that warrants timely identification due to its significant impact on treatment planning and long-term management.

Lymphoproliferative malignancies associated with CMMRD are linked to poor outcomes due to resistance to standard chemotherapy and increased risk of treatment-related toxicity. Early genetic diagnosis, personalized treatment protocols, and family counseling are essential components of care for affected patients. Advances in immunotherapy and molecularly targeted prevention strategies offer hope, but further research is needed to improve outcomes in this challenging clinical population.

## Data Availability

The datasets presented in this study can be found in online repositories. The names of the repository/repositories and accession number(s) can be found in the article/supplementary material.
